# An Epidemiological Study of Neuropathic Pain Symptoms in Canadian Adults

**DOI:** 10.1155/2016/9815750

**Published:** 2016-03-30

**Authors:** Elizabeth G. VanDenKerkhof, Elizabeth G. Mann, Nicola Torrance, Blair H. Smith, Ana Johnson, Ian Gilron

**Affiliations:** ^1^School of Nursing and Department of Anesthesiology and Perioperative Medicine, Queen's University, Kingston, ON, Canada K7L 3N6; ^2^School of Nursing, Queen's University, Kingston, ON, Canada K7L 3N6; ^3^Ninewells Hospital and Medical School, University of Dundee, Dundee DD2 4DB, UK; ^4^Population Health Sciences, Ninewells Hospital and Medical School, University of Dundee, Dundee DD2 4DB, UK; ^5^Department of Public Health Sciences, Queen's University, Kingston, ON, Canada K7L 3N6; ^6^Departments of Anesthesiology & Perioperative Medicine and Biomedical & Molecular Sciences, Queen's University, Kingston, ON, Canada K7L 3N6

## Abstract

The reported prevalence of neuropathic pain ranges from 6.9% to 10%; however the only Canadian study reported 17.9%. The objective of this study was to describe the epidemiology of neuropathic pain in Canada. A cross-sectional survey was conducted in a random sample of Canadian adults. The response rate was 21.1% (1504/7134).* Likely* or* possible* neuropathic pain was defined using a neuropathic pain-related diagnosis and a positive outcome on the Self-Report Leeds Assessment of Neuropathic Symptoms and Signs pain scale (S-LANSS) or the Douleur Neuropathique 4 (DN4) Questions. The prevalence of* likely* neuropathic pain was 1.9% (S-LANSS) and 3.4% (DN4) and that of* possible* neuropathic pain was 5.8% (S-LANSS) and 8.1% (DN4). Neuropathic pain was highest in economically disadvantaged males. There is a significant burden of neuropathic pain in Canada. The low response rate and a slightly older and less educated sample than the Canadian population may have led to an overestimate of neuropathic pain. Population prevalence varies by screening tool used, indicating more work is needed to develop reliable measures. Population level screening targeted towards high risk groups should improve the sensitivity and specificity of screening, while clinical examination of those with positive screening results will further refine the estimate of prevalence.

## 1. Introduction

Neuropathic pain was recently redefined by the International Association for the Study of Pain as “pain caused by a lesion or disease of the somatosensory nervous system” [1] and graded as “possible,” “probable,” or “definite,” depending on the extent and results of neurological assessment [2, 3]. Early estimates of the prevalence of neuropathic pain based on physical examination in clinic populations ranged from 1% to 3% [4–7]. In the general population, previous estimates based on self-report ranged from 6.5% to 17.9% [8–13]. A recent systematic review of epidemiological studies on neuropathic pain suggests that the prevalence likely lies between 6.9% and 10% [14]. Neuropathic pain is a clinical entity [1], the diagnosis of which is based primarily on history and physical examination [15–19] and the exclusion of other possible diagnoses or types of pain. The impracticality of conducting clinical examinations in large population studies and the lack of a “gold standard,” in addition to the variety of screening tools and the way in which they are administered, contribute to the heterogeneity of estimates of neuropathic pain in the general population [14].

Neuropathic pain is often experienced in parts of the body which otherwise appear normal. It is characterized by features such as numbness, paresthesia, and allodynia [20], and it is generally nonresponsive to standard analgesics to treat chronic pain [21]. It is also associated with longstanding and severe pain [22] and with a burden of disease similar to that of other chronic conditions, including mental health conditions [23]. A multitude of conditions can cause neuropathic pain, including lumbar and cervical radiculopathy, painful diabetic neuropathy, HIV-related neuropathy and cancer-related neuropathic pain syndromes [17, 24–27]. Certain groups within the general population have been identified as having a higher burden of neuropathic pain, including women [8, 10–12, 28], older people [8, 10, 11, 28], those with less formal education [10, 11], manual workers or farmers [8], those unable to work [11], those living in rural residences [8], non-home owners [11], and those perceiving themselves as being economically disadvantaged [12]. There is inconsistent evidence on the relationship between marital status and neuropathic pain [8, 10, 11]. There is also insufficient evidence to suggest a regional pattern of neuropathic pain prevalence. Most studies have been conducted in Europe and the United Kingdom where rates are lower compared to the few reports from other parts of the world. In the Americas, 9.8% has been reported with clinical examination and 12.4% using self-report in Olmsted County, Minnesota, USA [29], and 10% in Brazil [13]. The only Canadian study, conducted in Alberta, reported a prevalence of 17.9%, considerably higher compared to reports in other parts of the world [12].

The purpose of this study was to describe the epidemiology of neuropathic pain in a pan-Canadian community sample. The specific objectives were to estimate the prevalence and characteristics of likely or possible neuropathic pain and to identify subgroups with a high burden of neuropathic pain.

## 2. Methods

This cross-sectional study was reviewed for ethical compliance and received approval from the Queen's University Health Sciences Research Ethics Board (HSREB). The requirement for written informed consent was waived by the HSREB. The polling company, SM Research (http://www.smres.com/), used the national telephone directory to provide a random sample of 8000 households in the ten Canadian provinces. This method provides each household with an equal opportunity to be included in the sample. At the time of sampling, approximately 80% of Canadian households were listed in the directory with 20% not covered because they did not have a landline or were unlisted. After the survey was complete, SM Research provided weights so the sample could be age- and sex-matched to the 2011 Canadian Census data [30]. Based on published prevalence estimates, it was established that a sample size of 4000 would result in 95% confidence intervals (CI) ranging from 0.6% for a prevalence of 3.3% to 1.2% for a prevalence of 17.9% [31].

### 2.1. Participants

Potential participants were sent a bilingual (French and English) cover letter explaining the study. Due to the restrictive cost of postage, a unilingual paper questionnaire based on most likely language (as identified by polling company SM Research) was included. This resulted in French questionnaires being sent to all potential participants residing in the province of Quebec and those identified outside of Quebec as likely being French by the polling company (SM Research). Participants were encouraged to contact the research team if they preferred a questionnaire in the alternate language. In addition, they were provided with a link to an online version of the questionnaire in both official languages. Participants completing online questionnaires were required to enter their unique identifying number to allow for tracking of responses and to prevent the inclusion of duplicate (paper and online) responses. By completing the questionnaire, consent was implied. The online questionnaire was created using Student Voice (http://www.studentvoice.com/). Each envelope contained a stamped, self-addressed return envelope. The first mail out occurred in November 2011. Response time was delayed due to a postal disruption just before the initial mail out; therefore a follow-up to nonresponders was delayed until May 2012, when fewer than 20 questionnaires per week were being returned.

### 2.2. Case Identification and Measurement Tools

Individuals with chronic pain were identified by affirmative answers to two screening questions: (i) Are you currently troubled by pain or discomfort, either all of the time or on and off? and (ii) Have you had this pain or discomfort for more than 3 months? These case identification questions are based on the International Association for the Study of Pain definition of chronic pain [32] and have been validated and used in previous studies of chronic pain [11, 33, 34]. Participants with positive responses to both questions were asked the following: How often are you bothered by this pain or discomfort (all the time, daily (but not the entire day), nearly daily, on and off)? The frequency categories were based on reports from previous studies [11, 12]. Participants were asked to identify the location of the most troublesome pain based on a numbered body manikin which included back; neck or shoulder; head, face, or teeth; stomach or abdomen; arms or hands; chest; hips; and legs or feet [35] and to identify all body sites at which they experienced pain. They were also asked if they had been diagnosed with any of the following common causes of pain: a surgical operation more than three months ago, back problems (such as a slipped disc, back surgery, or sciatica), diabetes, an accident that damaged a nerve, amputation of a limb, fibromyalgia, leg ulcers, shingles, cancer, chronic widespread pain, migraine, arthritis, and vulvodynia.

The Self-Report Leeds Assessment of Neuropathic Symptoms and Signs (S-LANSS) pain scale and the Douleur Neuropathique 4 (DN4) Questions, both of which have been validated in clinical populations with neuropathic pain, were used to screen for chronic pain with neuropathic characteristics [36, 37]. The S-LANSS consists of 5 symptom items and 2 self-examination items, with responses weighted to provide a score ranging from 0 to 24. A score of ≥12 is suggestive of pain with neuropathic characteristics [36]. The S-LANSS has a sensitivity of 74% and a specificity of 76% when compared to clinical examination [36]. The self-report version of the DN4 consists of 7 items related to symptoms. Participants are asked for a yes/no response to questions about quality of pain (burning, painful cold, and electric shocks) and its association with abnormal sensations (tingling, pins and needles, numbness, and itching). The summary score ranged from 0 to 7 with a score of ≥3 out of 7 being suggestive of chronic pain with neuropathic characteristics [37]. In order to be classified as neuropathic pain, an individual also had to report a* likely* (accident with nerve damage, amputation, and shingles) or* possible* (surgery more than 3 months ago, back problem, diabetes, and cancer) neuropathic pain diagnosis (the full list is available upon request). This self-report version of the DN4 has a sensitivity of 78% and a specificity of 81% [38].

Severity of neuropathic pain was measured using the Neuropathic Pain Scale (NPS), a series of 11-item numerical rating scales that address specific features of the pain experience (intensity, sharpness, hotness, dullness, coldness, sensitivity, and itch) and items measuring unpleasantness, timing, and surface and deep pain [39]. Median and interquartile range (IQR) were calculated for each item. Help seeking behavior was assessed using the Level of Expressed Need (LEN) questionnaire [40]. The LEN asks about the seeking of treatment and use of painkillers. The 4 questions of the LEN are summed to create a score ranging from 0 to 4, with 0 reflecting no treatment or painkillers and 4 representing maximum need (frequent use of painkillers and recent treatment sought). Participants were also asked, “What treatments or medications are you receiving for your pain?”

Participants were asked to report any diagnosed chronic health conditions including asthma; anxiety disorder (e.g., phobia, obsessive-compulsive disorder, or panic disorder); bowel disorder (e.g., Crohn's disease, ulcerative colitis, irritable bowel syndrome, or bowel incontinence); chronic bronchitis, emphysema, or chronic obstructive pulmonary disease; chronic fatigue syndrome; diabetes; heart disease (e.g., heart attack, congestive heart failure); hypertension or high blood pressure; mood disorder (e.g., depression, bipolar disorder, mania, or dysthymia); multiple chemical sensitivities; intestinal or stomach ulcers; stroke; and urinary incontinence. Sociodemographic characteristics captured included age, gender, smoking history, marital status, employment status, educational attainment, income, and home ownership. Age was categorized into approximate quartiles to match categories used in the Canadian Census data and thereby allow for extrapolation to the Canadian population. Age was also categorized by a median split for the purpose of the multivariable analysis. Income was categorized into approximate tertiles, again to allow for comparisons with Census data.

### 2.3. Data Analysis

Data quality checks were conducted using established methodology before merging datasets [41]. Every tenth questionnaire was checked and if an entry error was found every survey was checked until ten consecutive error-free surveys were reviewed [41]. Once the quality check was completed, online survey data were downloaded into a Microsoft Excel file and merged with the manually entered data which were also in Microsoft Excel. Descriptive statistics were used to summarize characteristics of the study sample. Prevalence estimates were age- and sex-weighted to the 2011 Canadian Census data [30]. For logistic regression and decision tree analyses, weights were scaled down (weight/5000) to avoid excessively narrow estimates of precision. The prevalence of chronic pain with neuropathic characteristics was calculated in three ways: as the percentage with a positive score on the (i) S-LANSS (≥12), (ii) the DN4 (≥3), and (iii) on both tools. The prevalence of neuropathic pain was calculated as the percent with chronic pain with neuropathic characteristics and with a* likely* or* possible* neuropathic pain diagnosis. The clinical and pain characteristics of individuals with neuropathic pain were described using percentages and measures of central tendency. Unadjusted and adjusted logistic regression analyses were conducted to examine the relationship between sociodemographic characteristics and neuropathic pain. Decision tree analysis was conducted to identify the subgroups reporting the highest percent of individuals with neuropathic pain. Variables were dichotomized to ensure adequate cell frequencies. The percentage of the total population within each subgroup and the percentage of individuals with neuropathic pain within each subgroup are reported. Risk estimates (standard error) were calculated as a measure of the tree's predictive accuracy. The risk estimate is the proportion of cases incorrectly classified after adjustment for prior probabilities and misclassification costs (SPSS® online help Decision Tree Option). The percent of missing items was calculated for the S-LANSS, DN4, and NPS. To minimize the effect of missing data on the screening tools, missing values were assigned a “0.” This would have resulted in a conservative total score for the S-LANSS and DN4. Analysis was conducted using IBM® SPSS version 22 [42].

## 3. Results

**Figure 1:**
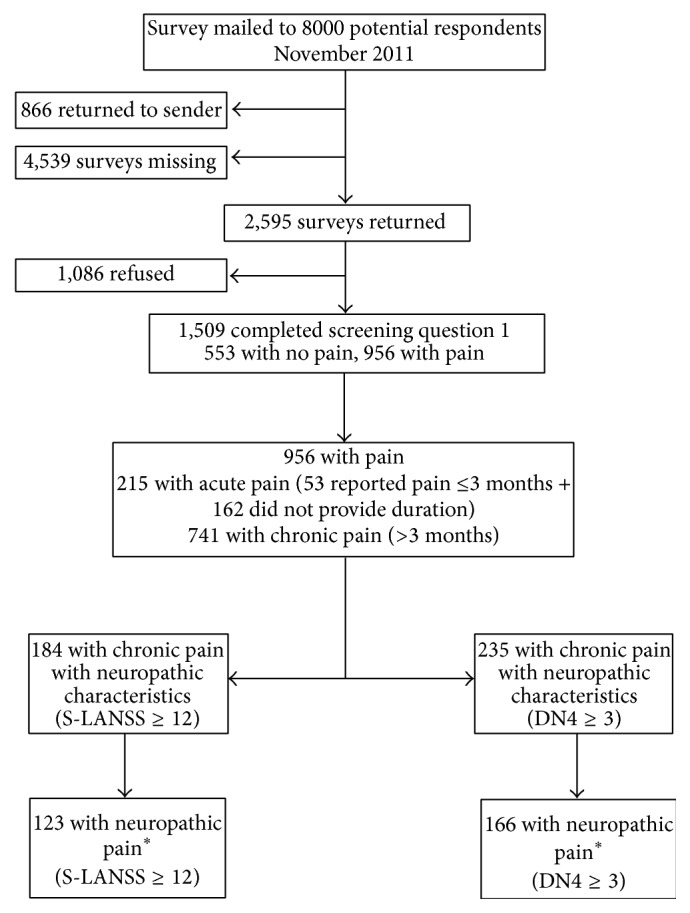
Flowchart of sample participants. Question 1: Are you currently troubled by pain or discomfort, either all the time or on and off? ^*∗*^Diagnosed with a likely or possible neuropathic pain condition and positive on the screening tool.

Of the 8,000 questionnaires, 866 (10.8%) were returned due to wrong address, 4,539 (56.8%) were not returned, 2,595 (32.4%) were returned, and 1,509 (18.9%) were at least partially completed (Figure 1). A postal strike delayed mailing of the questionnaires and may have affected the delivery and return of questionnaires even after the postal strike was over. The corrected response rate based on the proportion of questionnaires sent to the correct address and with the pain screening questions completed was 21.1% (1504/7134). Ninety-three percent (1395/1504 = 92.8%) of respondents completed paper questionnaires and 7.2% (109/1504) completed online questionnaires. The percent of missing data ranged from 2.0% to 5.7% on the S-LANSS, 9.2% to 15.0% on the DN4, and 3.2% to 5.0% on the NPS. The percent of missing responses on potentially sensitive questions was also examined (e.g., household income (8.5% missing) and home ownership status (2.9% missing)). Respondents completing paper questionnaires were significantly older (mean = 58 SD = 14 versus mean = 50 SD = 13, *t*-test = 5.5, and *p* < 0.01), reported lower household income, were retired, and had less education than those completing online questionnaires.

The prevalence of likely neuropathic pain was 1.9% (margin of error (ME) = 0.7, sample *n* = 33, population *N* = 504,137) on the S-LANSS and 3.4% (ME = 0.9, *n* = 48, *N* = 894,618) on the DN4. The prevalence of possible neuropathic pain was 5.8% (ME = 1.1, *n* = 90, *N* = 1,526,214) on the S-LANSS and 8.1% (ME = 1.3, *n* = 118, *N* = 2,132,903) on the DN4. The remaining results focus on the combined prevalence of likely or possible neuropathic pain.

Table 1 includes the sociodemographic and comorbidity characteristics for the sample (*n* = 1504) and the age- and sex-weighted population of Canadian adults (*N* = 26,423,076). The largest proportions of participants were from Ontario (38.3%) and Quebec (24.0%), which is consistent with the distribution by province captured in the 2011 Canadian Census (Table 1). Sociodemographic and clinical characteristics stratified by* likely* or* possible* neuropathic pain using the S-LANSS and the DN4 are also reported in Table 1. The overall prevalence of neuropathic pain was higher on the DN4 than on the S-LANSS and this pattern persisted for most of the sociodemographic and clinical characteristics. However, in some cases the prevalence was much higher than would be expected on the DN4 compared to the S-LANSS (e.g., male 13.6% versus 6.5%, 18–39-year-olds 9.6% versus 3.5%, stroke 30.3% versus 13.5%, anxiety 36.1% versus 16%, and mood disorder 28.9% versus 16.8%).

Of the total population represented by this sample, 4.9% reported having had an accident with nerve damage (Table 2). Of the 4.9%, 31.4% had a positive screen for neuropathic pain characteristics on the S-LANSS and 51.6% on the DN4. The prevalence of neuropathic pain by all sites and the most troublesome site is reported in Table 3. The highest prevalence of neuropathic characteristics was reported in lower extremities (S-LANSS 73.1%, DN4 78.4%), back/buttocks (S-LANSS 68.9%, DN4 73.0%), and upper extremities (S-LANSS 64.7%, DN4 61.1%); however the most troublesome site was the back/buttocks (S-LANSS 27.9%, DN4 32.6%). Approximately half of the population with neuropathic pain reported background pain all of the time with occasional flare-ups some of the time (S-LANSS 51.7%, DN4 55.8%). The median pain intensity of neuropathic pain was 6.0/10 (IQR 5.0–8.0) (S-LANSS) and 7.0/10 (5.0–8.0) (DN4). The highest pain ratings were described as sharp (both tools 7.0 (4.0–8.0)), dull (both tools 5.0 (3.0–7.0)), and hot (S-LANSS 4.0 (2.0–6.0), DN4 3.0 (1.0–5.0)). Deep pain intensity was rated as a median of 7.0 (5.0–8.0) (both tools) and surface pain was rated as 5.0 (3.0–7.0) (S-LANSS) and 4.0 (2.0–6.0) (DN4). Over one-third of those with neuropathic pain reported the maximum Level of Expressed Need; that is, treatment was sought and painkillers were used frequently and recently (S-LANSS 39.7%, DN4 40.3%). The most common forms of treatment for pain were prescription opioid and/or prescription anti-inflammatory medication (S-LANSS 27.1%, DN4 24.8%) or over the counter analgesics (S-LANSS 26.6%, DN4 23.7%).

The unadjusted and adjusted odds ratios (OR) and 95% confidence intervals (CI) for sociodemographic characteristics associated with neuropathic pain are reported in Table 4. In the adjusted model, the factors significantly associated with increased odds of neuropathic pain (S-LANSS) were income (<$50,000 OR = 4.59, 95% CI 3.26–6.46; $50,000–$99,999 OR 1.58, 95% CI 1.13–2.21; and ref ≥$100,000), being unemployed (OR = 1.89, 95% CI 1.43–2.46, and ref = working full- or part-time), and being a past smoker (OR = 1.86, 95% CI 1.47–2.35, and ref = never smoked). Being married decreased the odds of neuropathic pain (OR = 0.56, 95% CI 0.44–0.72). Similar findings existed for the DN4 analysis: income (<$50,000 OR = 3.44, 95% CI 2.59–4.58; $50,000–$99,999 OR 2.16, 95% CI 1.65–2.83; and ref ≥$100,000), being unemployed (OR = 3.05, 95% CI 2.42–3.84, and ref = working full- or part-time), and being a current smoker (OR = 2.39, 95% CI 1.88–3.05, and ref = never smoked). Being female had increased odds for neuropathic pain in the S-LANSS model (OR = 1.46, 95% CI 1.17–1.81) and decreased odds in the DN4 model (OR = 0.60, 95% CI 0.50–0.72). Contrary results were also found for age between the two outcomes. Mode of survey (paper versus online) administration was significant in bivariate analysis. It was not included in the multivariable model due to the low number of individuals who completed the online component (*n* = 109), which would have affected the stability of the analysis, and due to the potential for confounding because of its relationship with both the independent factors (i.e., age, income) and the outcome.

**Figure 2:**
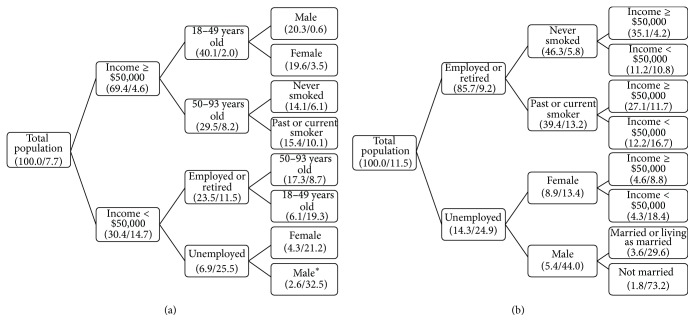
Decision tree analysis-percent of population in each subgroup and the percent with neuropathic pain within the respective subgroup (*N* = 26,423,339). (a) Probable or possible neuropathic pain condition and screening positive on the SLANSS (Self-Report Version of the Leeds Assessment of Neuropathic Symptoms and Signs). Risk estimate (standard error) = 0.077 (.004). (b) Probable or possible neuropathic pain condition and screening positive on the DN4 (Douleur Neuropathique 4). Risk estimate (standard error) = 0.106 (0.000). Numbers in brackets represent % of total population and % with neuropathic pain within that subpopulation e.g., ^*∗*^Male (2.6/32.5) = 32.5% of males who were unemployed with an annual income <$50,000 reported neuropathic pain and this represents 2.6% of the total population of Canada. All findings have an adjusted *p* value <0.001. Growing method was CHAID.

When all significant sociodemographic main effect terms were included in the decision tree analysis, the highest prevalence of neuropathic pain (S-LANSS) was found in males who were unemployed with an income <$50,000 per year (32.5%, *N* = 226,926) (Figure 2(a)). The lowest prevalence was found in males aged 18 to 49 years with an income ≥$50,000 (0.6%, *N* = 33,740). This represents a relative risk of 54.2 (32.5/0.6) in males who were unemployed with an income <$50,000 per year versus males aged 18 to 49 years with an income ≥$50,000. In the DN4 decision tree analysis, the highest prevalence of neuropathic pain was found in unmarried males who were unemployed (73.2%, *N* = 344,881) (Figure 2(b)). The lowest prevalence was found in individuals with an income ≥$50.000 who had never smoked and were employed or retired (4.2%, *N* = 389,816). This represents a relative risk of 17.4 (73.2/4.2).

## 4. Discussion

This is the first study to use two validated screening tools to report the prevalence of neuropathic pain in a general population. The prevalence of* likely or possible* neuropathic pain was 7.7% (S-LANSS) and 11.5% (DN4). Males were twice as likely to report neuropathic pain using the DN4 compared to the S-LANSS (13.6% vs. 6.5%). Less than 10% used a combination of medication and nonmedication therapy. Males who were economically disadvantaged had the highest burden of neuropathic pain.

Chronic pain with neuropathic characteristics, that is, based on the results of the screening tool alone, not taking into account diagnosis, is lower in our study (11.8% S-LANSS, 16.1% DN4), compared to the one other Canadian study (17.9% DN4) [12]. In the other Canadian study by Toth et al., respondents were asked to complete the DN4 if they reported “daily or near-daily pain” perhaps resulting in an overestimate of neuropathic pain [12]. Our prevalence of neuropathic pain (taking diagnosis into account) of 7.7% (S-LANSS) is consistent with the prevalence of chronic pain with neuropathic characteristics (not accounting for diagnosis) reported in the UK (UK 8%) [11], but the DN4 based estimate (11.5%) is higher compared to reports in Germany (6.5%) [10], France (6.9%) [8], and Morocco (10.6%) [9].

Neuropathic pain was associated with low income, unemployment, smoking, and being unmarried. Other studies have reported similar findings [8, 11, 12]. Unique to this study is the fact that males had a higher prevalence of neuropathic pain than females (DN4). In addition, there was a high burden in males who were economically disadvantaged. This suggests potential deprivation-related factors, while the high prevalence of back disorder diagnoses suggests certain occupations involving manual labour that may increase the risk for neuropathic pain.

Neuropathic pain was associated with hypertension, diabetes, bowel disorders, and mood disorders. The limited quality and completeness of data on comorbidities have been well documented [43]; however self-report of comorbidities has been shown to be comparable to documentation in the medical record [44, 45]. Several of our findings are consistent with the literature. Hypertension and bowel disorders have been associated with chronic pain conditions in other studies [46–51]. Individuals with diabetes may experience diabetic neuropathy, and this group is also thought to have a higher prevalence of other neuropathic pain conditions due to a “double crush” neuropathic susceptibility [52]. Finally, the high correlation between chronic pain and mood disorders has been documented [53].

Using the grading system for neuropathic pain [1, 3],* likely* neuropathic pain was consistent with clinical prevalence [4–7], and* likely* and* possible* neuropathic pain was consistent with population prevalence [14]. Related to this is the limited sensitivity and specificity of existing screening tools. A proportion of respondents who were positive for chronic pain with neuropathic characteristics had a pain condition that was* unlikely* neuropathic. Similar findings have been reported with the DN4: fibromyalgia (93% Toth et al. 2009 [12] versus 51% our study), migraine (36% versus 17%), osteoarthritis (11% versus 11%), and vulvodynia (60% versus 68%) [12]. False positive results are partially related to features (e.g., burning, shooting, and allodynia) common to DN4 and S-LANSS not being completely exclusive to neuropathic conditions. However, a higher proportion of* likely* neuropathic conditions were positive on the screening tools than were conditions that were* unlikely* neuropathic.

Strengths of this study include the use of two screening tools and the inclusion of a neuropathic pain-related diagnosis to measure the prevalence. This permitted a gradation of estimates ranging from chronic pain with neuropathic characteristics to* likely* neuropathic pain. An additional strength is the national scope of the study; inclusion of all provinces in Canada; and the use of bilingual questionnaires. Due to low response rates, territories were not included, nor were questionnaires available in languages spoken by the First Nations people of Canada, which likely contributed to the lack of response in the territories. Nor have the current tools been validated in these cultures or languages. A limitation was provision of the initial questionnaire in the assumed first language of the potential participant, as identified by the polling company. This may have deterred individuals who required a questionnaire in the other official language. However, there was no differential response by language given that the ratio of French to English questionnaire completion was reflective of language as reported on the Canadian Census. A further strength was that random sampling was used for selecting the study sample and weighting the final sample to the age and sex of the population. With the exception of being older and having lower income, the age- and sex-weighted demographic characteristics of the study sample were similar to the Canadian Census data.

A limitation of our study was the inability to validate the presence of neuropathic pain through clinical examination. We used the best available methodology for identifying neuropathic pain in the community, which relies on a combination of screening tools and relevant diagnosis [54]. However, if respondents incorrectly cited a neuropathic pain diagnosis and if they were positive on a tool, we would have considered them as having neuropathic pain, leading to an overestimate of prevalence. Conversely, if respondents with a true neuropathic pain diagnosis did not provide a pain diagnosis, despite having a positive score on the screening tools, they would have been considered nonneuropathic, leading to an underestimation of prevalence. Not all back problems are neuropathic [55]; however we included all individuals reporting a diagnosed back problem in our definition. Given the high prevalence of back problems in our study (19.7%), this may have resulted in an overestimate of prevalence of neuropathic pain. When individuals with back problems were excluded, the prevalence of neuropathic pain dropped to 4.1% (S-LANSS) and 5.3% (DN4). We can also assess some degree of underestimation. For example, of respondents who reported chronic pain but did not provide a pain diagnosis, 18 were positive on the S-LANSS and 23 were positive on the DN4. If all of these respondents had a true neuropathic pain diagnosis, the prevalence would increase to 9.2% (S-LANSS) and 13.4% (DN4).

The difference in prevalence of chronic pain with neuropathic characteristics between the two screening tools may be related to design and content of the questionnaires. Although there are several specific neuropathic pain characteristics common to both the S-LANSS and DN4, there are also differences in these screening tools [56]. For example, “painful cold,” “numbness,” and “itching” are included in the DN4 but not the S-LANSS, whereas “pain evoked by light touch” and autonomic “colour changes” are only present in the S-LANSS. In this study, median scores for the NPS items for both “cold” and “itching” pain were zero; both of these pain characteristics are included in DN4 but not in S-LANSS. Future research is needed to explore the implications of these differences. Five distinct subtypes of neuropathic pain based on symptoms and sensory profiles have been identified, alongside different frequencies in different neuropathic conditions [57]. It is unclear whether these have implications for management and response to treatment but as more evidence emerges, subdividing the epidemiology of neuropathic pain may be explored [58]. Missing data may also explain the discrepancy in prevalence. There was a higher proportion of missing responses for the 7 items on the DN4 (9.2%–15.0%) compared to the 7 items on the S-LANSS (2.0%–5.7%). This may have been due to respondent fatigue given that the DN4 followed the S-LANSS in the order of the questions; however the NPS followed the DN4 and the proportion of missing responses for the NPS was lower than that for the DN4 and similar to that of the S-LANSS. A more likely explanation is the way in which the questions are posed on the DN4, leading respondents to not answering when the response is negative.

An important limitation of our study was the low response rate which may have led to response bias, given that individuals with chronic pain may be more likely to respond to a survey about chronic pain [59]. The low response rate was partially due to a postal strike, which may have resulted in delayed delivery and lost questionnaires; however this technical difficulty unlikely led to response bias. A small proportion of respondents (8.5%) completed online questionnaires, which may have had minimal effect on the findings for the S-LANSS in particular, where a higher proportion of respondents were positive for chronic pain with neuropathic characteristics on paper versus online questionnaires. However, respondents who completed paper questionnaires were older and had lower income, factors also associated with neuropathic pain. Therefore survey mode was likely a confounder and thus was not included in multivariable analyses. The low response rate in this study is consistent with response rates for survey research, which are declining in general [11, 60, 61], and although it may influence our estimates of prevalence, it should have minimal effect on risk factors associated with neuropathic pain.

This study provides evidence of a significant burden of neuropathic pain in the general population of Canadians. The highest burden was in males who were economically disadvantaged. Prevalence varied by screening tool, indicating more work is needed to develop reliable measures. Better training of primary care practitioners and community based clinical studies might help refine the precision of the estimates. Given the major resource implications associated with population level screening, screening could be targeted towards groups known to be at increased risk for neuropathic pain. Not only would this improve the sensitivity and specificity of screening, but also clinical examination to further define the condition would be more manageable. Finally, prospective studies are needed to shed more light on the etiology and incidence of neuropathic pain.

## Figures and Tables

**Table 1:** Sociodemographic and comorbidity characteristics of the study sample weighted to total population of Canadian adults (26,423,076) and stratified by *likely* or *possible* neuropathic pain status.

	Sample (*n* = 1504)	Sample weighted	2011 Census^a^	*Likely* or *possible* NP (S-LANSS)^b^ (population *N* = 2,030,351)	*Likely* or *possible* NP (DN4)^c^ (population *N* = 3,027,521)
Mean age (standard deviation)	57.6 (14.2)	51.6 (14.7)	NA	55.0 (13.1)	51.5 (14.8)

	*n*	%	%	%	%

Gender					
Male	818	48.5	48.7	6.5	13.6
Female	686	51.5	51.3	8.8	9.5
Age groups					
18–39 years	162	20.5	36.0	3.5	9.9
40–49 years	220	28.8	18.8	7.5	12.3
50–65 years	660	30.5	26.5	10.8	12.2
66–93 years	462	20.2	18.6	7.6	10.7
Region					
British Columbia	173	13.4	14.6	9.5	14.4
Alberta	123	10.7	11.5	2.7	11.9
Saskatchewan, Manitoba	100	6.5	7.0	8.6	11.3
Ontario	647	38.3	41.7	6.6	9.1
Quebec	403	24.0	25.7	8.2	11.7
Eastern provinces^d^	58	7.2	7.6	15.0	17.2
Education					
High school or less	364	22.6	45.7	9.6	17.3
CEGEP^e^, trade or professional certificate, or others	406	31.4	29.1	9.2	12.8
Some university, degree, or graduate degree	585	42.0	25.2	5.7	8.2
Own/mortgage home					
Yes	1308	86.0	69.4	6.9	9.6
No	196	14.0	30.6	12.3	23.0
Income					
<$50,000	567	32.0	40.1	14.1	18.0
$50,000–$99,999	557	36.9	33.2	5.6	11.2
$100,000+	380	31.1	25.9	3.6	5.0
Marital status					
Married or living as married	1077	70.3	57.7	7.7	10.6
Single, widowed, separated, or divorced	427	29.7	42.3	7.6	13.5
Employment status					
Working full- or part-time	774	63.0	60.9^f^	5.6	8.4
Retired	522	22.7	NA^g^	8.6	11.6
Looking for, incapable of, or not looking for work	208	14.3	NA	15.6	24.9
Smoking status					
Never smoked	710	52.7	NA	5.4	7.6
Previously smoked	577	31.7	NA	11.4	14.6
Current/occasional smoker	188	14.2	NA	8.3	19.6
Comorbid conditions					
Stroke	12	0.6	NA	13.5	30.3
Gastrointestinal condition	32	2.2	NA	22.4	22.4
Respiratory (not asthma)	48	2.5	NA	13.3	19.7
Anxiety	56	4.7	NA	16.0	36.1
Heart disease	68	3.1	NA	21.6	24.2
Mood disorder	82	5.7	NA	16.8	28.9
Bowel disorder	85	5.6	NA	26.5	22.2
Asthma	99	7.9	NA	10.0	12.4
Diabetes	130	6.1	NA	28.8	33.4
Hypertension	307	16.0	NA	17.2	24.7
Language of questionnaire					
English	1091	74.7	56.9/85.0^h^	7.6	11.5
French	411	25.3	21.3/30.1^h^	7.9	11.1
Survey mode					
Paper	1395	91.5	NA	6.5	11.4
Online^i^	109	8.5	NA	8.8	11.8

^a^Statistics Canada. 2011 National Household Survey. Based on Canadian population aged 15 and older (except age and sex which are based on age 18 and over).

^b^Likely or possible neuropathic pain diagnosis and ≥12 on the Self-Report Leeds Assessment of Neuropathic Symptoms and Signs.

^c^Likely or possible neuropathic pain diagnosis and ≥3 on the Douleur Neuropathique 4.

^d^Eastern provinces include New Brunswick, Nova Scotia, Prince Edward Island, and Newfoundland and Labrador.

^e^Collège d'Enseignement Général et Professionnel (General and Vocational College).

^f^Employment rate in 2011.

^g^Data not available.

^h^Mother tongue/working knowledge.

^i^Includes 2 questionnaires completed via telephone.

**Table 2:** Percent of respondents with pain-related diagnoses who were screened positive for neuropathic characteristics on the S-LANSS and the DN4^a^.

	Total		Diagnosed with pain condition	
	Sample (*n* = 1504) *n* (%)	Population (*N* = 26,423,076) %	S-LANSS ≥ 12^b^ (*N* = 3,128,817) %	DN4 ≥ 3^b^ (*N* = 4,264,722) %
*Likely neuropathic diagnosis*				
Accident with nerve damage	72 (4.8)	4.9	31.4	51.6
Shingles	32 (2.1)	1.6	29.0	48.0
Amputation	3 (0.2)	0.2	51.9	100
*Possible neuropathic diagnosis*				
Back	297 (19.7)	19.2	19.6	32.3
Surgery	123 (8.2)	8.8	33.5	40.8
Diabetes	104 (6.9)	4.9	34.3	39.4
Cancer	31 (2.1)	1.5	37.1	36.6
*Unlikely neuropathic diagnosis*				
Osteoarthritis	239 (15.9)	12.6	9.1	11.0
Arthritis—osteoarthritis or rheumatoid	114 (7.6)	7.0	25.9	17.3
Migraine	98 (6.5)	6.7	17.6	17.4
Rheumatoid arthritis	59 (3.9)	3.2	19.9	12.5
Chronic widespread pain	39 (2.6)	2.5	25.3	56.4
Fibromyalgia	35 (2.3)	1.9	37.5	50.6
Leg ulcers	6 (0.4)	0.3	0.0	0.0
Vulvodynia	4 (0.3)	0.2	0.0	67.6

^a^Individuals could select more than 1 condition.

^b^The percentage screening positive for possible neuropathic diagnosis excludes those with a concurrent likely neuropathic diagnosis. The percentage screening positive for unlikely neuropathic diagnosis excludes those with a concurrent likely or possible neuropathic diagnosis. S-LANSS = Self-Report Leeds Assessment of Neuropathic Symptoms and Signs; DN4 = Douleur Neuropathique 4.

**Table 3:** Characteristics and management of neuropathic pain (sample weighted to general population).

	Likely or possible NP (S-LANSS)^a^ (*N* = 2,030,351)	Likely or possible NP (DN4)^b^ (*N* = 3,027,521)
	%	%

*All pain sites* ^c^		
Lower extremities	73.1	78.4
Back/buttocks	68.9	73.0
Upper extremities	64.7	61.1
Neck	33.6	22.9
Chest/abdomen	31.1	24.2
Head	17.3	15.6
*The most troublesome pain site*		
Back/buttocks	27.9	32.6
Upper extremities	9.8	11.6
Lower extremities	15.8	14.1
Head/neck	5.8	4.2
Chest/abdomen/groin	3.3	2.4
More than 1 site	27.9	26.4
No site identified	9.1	8.4
*Timing of pain*		
All the time and flare-ups	51.7	55.8
Single type of pain all the time	28.7	23.4
Single type of pain sometimes	19.6	20.9

	Median (IQR)	Median (IQR)

*Neuropathic pain scale scores*		
Intensity	6.0 (5.0–8.0)	7.0 (5.0–8.0)
Sharp	7.0 (4.0–8.0)	7.0 (4.0–8.0)
Hot	4.0 (2.0–6.0)	3.0 (1.0–5.0)
Dull	5.0 (3.0–7.0)	5.0 (3.0–7.0)
Cold	0.0 (0.0–2.0)	0.0 (0.0–2.0)
Sensitive	3.0 (0.0–7.0)	1.0 (0.0–6.0)
Itchy	1.0 (0.0–5.0)	0.0 (0.0–4.0)
Unpleasant	6.0 (5.0–8.0)	6.0 (5.0–8.0)
Deep	7.0 (5.0–8.0)	7.0 (5.0–8.0)
Surface	5.0 (3.0–7.0)	4.0 (2.0–6.0)
*Level of Expressed Need*	***%***	***%***
Level 0	7.9	8.1
Level 1	9.1	10.0
Level 2	28.6	29.2
Level 3	14.7	12.4
Level 4	39.7	40.3

	%	%

*Treatments or medications*		
Prescription opioids/anti-inflammatories	27.1	24.8
Over the counter analgesics	26.6	23.7
Nonmedication techniques	8.7	17.6
Combination therapy (medication and nonmedication)	7.9	7.0
Antiseizure or antidepressants	6.7	5.5
Others	13.0	11.3
None/missing response	10.0	10.2

^a^Likely or possible neuropathic pain diagnosis and ≥12 on the Self-Report Leeds Assessment of Neuropathic Symptoms and Signs.

^b^Likely or possible neuropathic pain diagnosis and ≥3 on the Douleur Neuropathique 4.

^c^Respondents could choose more than one site.

**Table 4:** Unadjusted and adjusted odds (95% CI) of *likely* or *possible* neuropathic pain using the S-LANSS and the DN4.

	S-LANSS^a^		DN4^b^	
	Unadjusted OR (95% CI)	Adjusted OR (95% CI)	Unadjusted OR (95% CI)	Adjusted OR (95% CI)
Gender				
Male	1.0	1.0	1.0	1.0
Female	1.39 (1.13–1.71)	1.46 (1.17–1.81)	0.67 (0.56–0.79)	0.60 (0.50–0.72)
Age				
18–49 years	1.0	1.0	1.0	1.0
50–93 years	1.70 (1.38–2.10)	1.45 (1.13–1.85)	1.04 (0.87–1.23)	0.82 (0.67–1.02)
Marital status				
Married or living as married	1.0	1.0	1.0	Removed^c^
Single, widowed, separated, or divorced	0.98 (0.79–1.23)	0.56 (0.44–0.72)	1.32 (1.11–1.58)	
Education				
High school or less	1.0	Removed^c^	1.0	Removed^c^
CEGEP^d^, trade or professional certificate, or others	0.96 (0.73–1.25)		0.70 (0.56–0.87)	
Some university, degree, or graduate degree	0.57 (0.43–0.74)		0.43 (0.34–0.53)	
Employment status				
Working full- or part-time	1.0	1.0	1.0	1.0
Retired	1.60 (1.25–2.06)	0.65 (0.48–0.88)	1.43 (1.15–1.78)	1.09 (0.83–1.4)
Looking for, incapable of, or not looking for work	3.15 (2.46–4.03)	1.89 (1.43–2.46)	3.64 (2.97–4.47)	3.05 (2.42–3.84)
Income (Canadian)				
≥$100,000	1.0	1.0	1.0	1.0
$50,000–$99,999	1.59 (1.15–2.21)	1.58 (1.13–2.21)	2.41 (1.85–3.13)	2.16 (1.65–2.83)
<$50,000	4.45 (3.31–5.97)	4.59 (3.26–6.46)	4.19 (3.25–5.41)	3.44 (2.59–4.58)
Smoking status				
Never	1.0	1.0	1.0	1.0
Previous	2.23 (1.79–2.79)	1.86 (1.47–2.35)	2.07 (1.71–2.52)	1.69 (1.37–2.07)
Current	1.57 (1.16–2.13)	1.34 (0.97–1.85)	2.95 (2.35–3.70)	2.39 (1.88–3.05)

^a^Likely or possible neuropathic pain diagnosis and ≥12 on the Self-Report Leeds Assessment of Neuropathic Symptoms and Signs.

^b^Likely or possible neuropathic pain diagnosis and ≥3 on the Douleur Neuropathique en 4.

^c^Variables with *p* < 0.10 removed using backward manual procedure.

^d^Collège d'Enseignement Général et Professionnel (General and Vocational College).

## Additional Points

This cross-sectional survey describes the epidemiology of neuropathic pain in a community sample of Canadians.* Likely* or* possible* neuropathic pain was defined using a relevant diagnosis and a positive screen on the Self-Report Leeds Assessment of Neuropathic Symptoms and Signs (S-LANSS) pain scale or the Douleur Neuropathique 4 (DN4) Questions. Prevalence of* likely* neuropathic pain was 1.9% (S-LANSS) and 3.4% (DN4). Prevalence of* possible* neuropathic pain was 5.8% (S-LANSS) and 8.1% (DN4). Males who were economically disadvantaged had the highest burden of neuropathic pain. Prevalence varied by screening tool, indicating more work is needed to develop reliable measures.
